# Why diversity among participants in clinical studies is not always preferable: the case for small, exploratory studies

**DOI:** 10.1186/s12916-023-03151-8

**Published:** 2023-11-24

**Authors:** Anne-Marie Ellegaard, Filip Krag Knop

**Affiliations:** 1 Center for Clinical Metabolic Research, Copenhagen University Hospital – Herlev and Gentofte, Hellerup, Denmark; 2 Steno Diabetes Center Copenhagen, Herlev, Denmark; 3 Department of Clinical Medicine, Faculty of Health and Medical Sciences, University of Copenhagen, Copenhagen, Denmark; 4 Novo Nordisk Foundation Center for Basic Metabolic Research, Faculty of Health and Medical Sciences, University of Copenhagen, Copenhagen, Denmark

**Keywords:** Diversity, Small exploratory studies, Clinical research, Participant population

## Background

Undoubtedly, all sexes, races, ethnicities, and sexual orientations need representation in clinical trials. To increase health in a diverse population, the medical research supporting the development of new and existing medical therapies and diagnostics needs to be investigated in subsets of the target end users.

Within the last decade, the focus on diversity in pre-clinical and clinical research has spread. Journals, governmental agencies, and funding bodies increasingly require that relevant groups are included in the (planned) research before publication or approval. This has led to health benefits for less favoured groups; e.g. in 2018, the Food and Drug Administration (FDA) in the USA required the manufacturers of zolpidem-containing insomnia drugs to reduce the recommended dose for women to half of that for men because new data—that had not been available during drug development 20 years previously—showed a slower drug metabolism in women than in men [1]. Currently, the FDA is working on implementing requirements for a diversity plan for late-stage clinical research [2]. This is good news for the health of under-represented groups, such as the elderly and different ethnic groups [3].

## Main text

While we welcome the increasing number of initiatives to diminish unbalanced diversity in clinical trials, we would like to highlight situations where diversity is not necessarily desirable. In our research centre, we conduct—among other studies—small, exploratory, physiology studies. These studies can be difficult to power properly since the exploratory nature involves uncertainty regarding the variance of the data points. Thus, increasing the diversity of the study population in this type of study might increase the noise-to-signal ratio uncontrollably and unnecessarily. To compensate for an increased noise, more participants would be needed, thus, demanding more time and resources spent on the study, in turn requiring more money raised, and most importantly, more participants enduring the procedures and potentially associated discomfort related to such studies. In our opinion, this would be a waste of resources.

The point of the small, exploratory studies that we conduct is to test a scientific hypothesis in a limited sample size, which can then be followed up by larger studies if the initial study shows relevant results. If such exploratory studies were required to include four times the number of participants (younger and older, and of both biological sexes), very few would be performed given the limited availability of resources and the increasing practical and administrative work associated with such studies. However, it is important to highlight that recruiting participants from a selected group potentially may result in biased or incomplete findings, which limits their generalizability. This is not a problem in itself, but authors and reviewers of publications of early-phase clinical trials should address the limitations associated with this issue. Hence, we recommend that authors clearly state the following points in their publications, if applicable:

1. The nature of the research (pilot, exploratory, small scale)
2. Whether the participants were recruited among a relevant population for the (patho)physiology, intervention, or diagnostic under investigation (including the recruitment of relevant controls)
3. Which groups to include in later stage clinical trials to appropriately cover all relevant groups whom this research concerns
4. Whether the findings of the study are generalizable to the wider population

In our experience, an increasing number of reviewers for scientific journals question and criticise the limited diversity of some of our small-scale whole-body human (patho)physiology studies. We expect this to be a consequence of the generally raised awareness of diversity in phase 3 clinical trials. However, in the context of small, explorative physiology studies, we find the critique inappropriate and misunderstood for the reasons laid out above. Thus, we believe that by addressing the inherent issues of the limited diversity among participants in small, exploratory trials in publications authors can highlight both the merits and limitations of such studies more appropriately.

## Conclusion

In conclusion, as much as the increased focus on diversity in participant populations of phase 3 and 4 clinical trials in general is long overdue, we advocate for a less strict approach towards diversity in small, exploratory studies taking the nature of this type of study into account.

## Data Availability

Not applicable.

## Abbreviation

FDA: Food and Drug Administration
